# Epidemiology, Risk Factors and Genotypes of HBV in HIV-Infected Patients in the Northeast Region of Colombia: High Prevalence of Occult Hepatitis B and F3 Subgenotype Dominance

**DOI:** 10.1371/journal.pone.0114272

**Published:** 2014-12-02

**Authors:** Henry Bautista-Amorocho, Yeny Zulay Castellanos-Domínguez, Laura Andrea Rodríguez-Villamizar, Sindi Alejandra Velandia-Cruz, Jeysson Andrey Becerra-Peña, Ana Elvira Farfán-García

**Affiliations:** 1 University of Santander (UDES), Bacteriology and Clinical Laboratory Program, Department of Health Sciences, CliniUDES Research Group, Laboratory for Biomedical and Biotechnological Research (LBBR) Bucaramanga, Santander, Colombia; 2 Industrial University of Santander (UIS), Department of Public Health, School of Medicine, Research Group on Demography, Public Health and Health Systems (GUINDESS), Bucaramanga, Santander, Colombia; University of Cincinnati College of Medicine, United States of America

## Abstract

**Introduction:**

Chronic hepatitis B virus (HBV) infection is an increasing cause of morbidity and mortality in human immunodeficiency virus (HIV)-infected individuals. HIV-positive patients are commonly co-infected with HBV due to shared routes of transmission.

**Objectives:**

Our aim was to determine the risk factors, prevalence, genotypes, and mutations of the Surface S gene of HBV, and occult hepatitis B infection (OBI) among patients infected with HIV in a northeastern Colombian city.

**Methods:**

A cross-sectional study was conducted with 275 HIV-positive patients attending an outpatient clinic in Bucaramanga, Colombia during 2009–2010. Blood samples were collected and screened for serological markers of HBV (anti-HBs, anti-HBc and HBsAg) through ELISA assay. Regardless of their serological profile, all samples were tested for the HBV S gene by nested-PCR and HBV genotypes were determined by phylogenetic inference. Clinical records were used to examine demographic, clinical, virological, immunological and antiretroviral therapy (ART) variables of HIV infection.

**Results:**

Participants were on average 37±11 years old and 65.1% male. The prevalence of HIV-HBV coinfection was 12% (95%CI 8.4–16.4) of which 3.3% had active HBV infection and 8.7% OBI. The prevalence of HIV-HBV coinfection was associated with AIDS stage and ART treatment. Sequence analysis identified genotype F, subgenotype F3 in 93.8% of patients and genotype A in 6.2% of patients. A C149R mutation, which may have resulted from failure in HBsAg detection, was found in one patient with OBI.

**Conclusions:**

The present study found a high prevalence of HIV-HBV coinfection with an incidence of OBI 2.6-fold higher compared to active HBV infection. These findings suggest including HBV DNA testing to detect OBI in addition to screening for HBV serological markers in HIV patients.

## Introduction

Hepatitis B virus (HBV) and human immunodeficiency virus (HIV) are major public health problems, particularly in developing countries. Both viruses share risk factors and transmission routes which accounts for a high frequency of HIV-HBV coinfection [1]. Approximately, 35 million (32.2–38.8 million) individuals worldwide are HIV carriers, of which 3 to 6 million have chronic hepatitis B (CHB) for an estimated HIV-HBV coinfection incidence of 5–20% [2], [3].

In the last decade, mortality associated with acquired immunodeficiency syndrome (AIDS) and opportunistic infections has substantially decreased in regions with extensive use of antiretroviral therapy (ART). Nevertheless, liver disease has emerged as one of the top-five causes of morbi-mortality among people living with HIV [4], [5]. Compared to HBV mono-infection, HIV-HBV coinfection is associated with a fivefold increase in the risk of CHB progression, and a twofold increase in mortality due to end-stage liver disease [6]. ART classes, such as lamivudine and tenofovir, exhibit dual activity in co-infected patients by modifying the HBV serological profile and increasing drug resistance related to mutant HBV strains [7]–[9].

Screening for HBV infection consists of immunoenzymatic assays that detect surface antigens (HBsAg) and antibodies against the viral core (anti-HBc) [10]. Some individuals infected with HBV are HBsAg negative; a clinical condition known as occult HBV infection (OBI) [11]–[13]. These individuals are eventually diagnosed using molecular biology techniques for viral DNA isolation in blood or liver tissue. Unfortunately, in developing countries such as Colombia, molecular testing for HIV-HBV coinfection is not always performed.

A variety of hypotheses have been put forward to explain the mechanism of OBI: formation of HBsAg – antibodies against S antigen (anti-HBs) immune complexes, low levels of HBV DNA replication, mutations in the S gene immunogenic domain, and viral interference mediated by the hepatitis C virus [13]–[15]. Similar to the end stage of CHB, OBI can result in adverse clinical outcomes such as acute liver failure, cirrhosis or cellular hepatocarcinoma (CHC) [16]–[17].

The prevalence of HIV-HBV coinfection varies according to the burden of HBV infection across and within countries [18]. Studies in Colombia have indicated that HBV is endemic with regional variations (low, intermediate and high endemicity patterns) [19]. Epidemiological reports have shown an increase in the incidence of HBV from 3.1 cases per 100,000 inhabitants in 2008 to 4.8 in 2012 [20], [21]. Despite the implementation of public health strategies to reduce the burden of HIV and HBV in Colombia, both viral infections have increased over the last decade. To date, no studies have been conducted to establish the incidence of simultaneous coinfection of HIV with OBI, and HBV genotype distribution among HIV patients in Colombia.

The objective of this study was to determine the prevalence of HBV and OBI among patients living with HIV in northeast Colombia, a geographic region with low HBV endemicity. In addition, we aimed to identify genotypes, subtypes and mutations of the HBV S gene, and explore associations with clinical, virological and immunological HIV variables. Our purpose is to provide knowledge to support strategies for the prevention, surveillance and control of the burden of disease caused by HBV and HIV infections in Colombia.

## Material and Methods

### Study design and participants

A cross-sectional study with non-probabilistic sampling was conducted among individuals who attended an outpatient clinic for HIV patients in Bucaramanga (the capital city of Santander) from January 2009 to July 2010. The region of study comprised a catchment area of 30,537 km^2^ with approximately 2 million inhabitants living in the Department of Santander in northeast Colombia. We included patients who had been previously confirmed for HIV infection by western blot after two positive ELISA tests. Socio-demographic, epidemiological, and HIV-related clinical and laboratory data were collected from recent medical records using a standardized form.

### Serological tests and HBV infection definitions

Blood specimens were collected in sterile tubes and sera were frozen in small aliquots at −80°C. All samples were tested in triplicate for HBsAg, anti-HBs, and anti-HBc using commercial ELISA kits (Biokit, Spain). Active HBV infection was defined as a positive HBsAg test. Both positive anti-HBs and anti-HBc tests indicated a resolved infection. Anti-HBs positive status was an indicator of vaccine-induced immunity. Isolated anti-HBc was confirmed when an anti-HBc serological test was positive with no other positive biomarker. OBI was defined as the detection of HBV DNA by nested PCR and HBsAg negative results. HIV-HBV coinfection was defined as evidence of current exposure to HBV, including active HBV infection and OBI.

### HBV DNA purification and amplification of the S gene

DNA was isolated from 1 ml of serum from HIV positive patients with the QIAamp Ultra Sense Virus kit (QIAgen, USA) according to the manufacturer's instructions. A 581 bp fragment of HBV S gene (HBs) was amplified with an in-house nested PCR protocol and a Platinum Taq DNA polymerase High Fidelity kit (Invitrogen, USA). We used PrimerSelect software (Lasergene 11.0, DNASTAR, USA) to design two sets of primers derived from a conserved region of the HBV genome. The outer primers were HBS1 forward (position 87-110 nts, 5′–ACCCTGTTCCGACTATTGCCTCTC – 3′) and HBS2 reverse (position 1746-1726 nts, 5′ – CCCCCAGCTCCTCCCAGTCCT – 3′). The inner primers were HBS3 forward (position 131-154 nts, 5′ – AAGACTGGGGGCCCTGCTATGAAC – 3′) and HBS4 reverse (position 711-688 nts, 5′ – AGCCCTACGCACCACTGAACAAAT – 3′). First round amplification was performed in a volume of 50 µl, containing 2 mM MgSO_4_, 0.2 mM each dNTP, 1 U Platinum Taq DNA high fidelity enzyme, 0.2 µM of each primer and 10 µl of sample DNA. After an initial denaturation step at 94°C for 2 min, 35 amplification cycles were carried out with denaturation at 94°C for 1 min, annealing at 55°C for 30 s and elongation at 68°C for 2 min. The second PCR round was performed under the same conditions, except 2 µl of the first-round product were loaded into the reaction tube. To avoid false negative or false positive results, rigorous testing procedures were followed to prevent sample cross-contamination. Experiments were run with appropriate controls, and test results were considered valid only when obtained in triplicate.

### DNA sequencing and phylogenetic analysis

PCR products were analyzed by electrophoresis using 1.3% agarose gels. A 581 bp fragment was excised with a commercially available QIAquick Gel Extraction Kit (QIAgen, USA) according to the manufacturer's instructions. Nucleotide sequences were determined from both strands using primers HBS3 and HBS4, and the resulting DNA was directly sequenced by automated dideoxy sequencing with a genetic analyzer (Applied Biosystems, USA). HBV genotypes and subtypes were determined by phylogenetic analysis with a panel of reference sequences from genotypes A to H retrieved from GenBank. The sequences were aligned with the Clustal Wallis algorithm using MegAlign software (Lasergene 11.0, DNASTAR, USA). The Neighbor-Joining method was used to construct the phylogenetic tree based on reliability bootstrap analysis with 1000 replicates using MEGA6 software. Finally, evolutionary distances were computed with the Kimura 2-parameter method [22]–[24].

### Analysis of mutations in the S region

The presence of mutations in the “a” antigenic loop and the major hydrophilic region (MHR) were analyzed from amino acids 124–147 and 100–160 of HBsAg, respectively. Translated amino acid sequences were compared with at least 15 reference sequences of the same genotype obtained from GenBank [25].

### Statistical analysis

Statistical analysis was performed using Stata version 11.1 (StataCorp, USA). Departure from normality of continuous variables was examined using the Shapiro-Wilk test. The null hypothesis of no difference of categorical, and normal and non-normally distributed continuous data was assessed using Chi-square, t-student and U-Mann Whitney tests, respectively. *P* values less than 0.05 were considered statistically significant. A binomial multiple regression analysis was conducted to test the association between HIV-HBV coinfection prevalence and patient clinical characteristics while adjusting for potential confounding variables. Prevalence ratios (PR) were calculated with 95% confidence intervals (95% CIs) around the estimate.

### Ethics

The University of Santander (UDES) Ethics Committee board approved the study protocol. All patients were invited to participate by their own physician and gave written consent after receiving detailed information about the study. For minors, the child's legal guardian signed the informed written consent. All patient data was handled anonymously and recorded using a serial number. This study was conducted in compliance with the Colombian Medical Code of Ethics and performed in accordance with the ethical standards laid down in the 1964 Declaration of Helsinki.

## Results

A total of 275 HIV-infected patients were recruited. Demographic, epidemiological, virological and immunological characteristics of all patients are summarized in Table 1. The majority of participants were male (65.1%) of heterosexual orientation (65.8%), with a mean age of 37.4±11.4 years (range from 4 to 66 years). The mean CD4 count was 384 cells/mm^3^ (Interquartile range IQR =  200–533). Mean CD4 counts for mono-infection and HIV-HBV coinfection groups were 392.3 cells/mm^3^ and 323.0 cells/mm^3^, respectively. The median total HIV viral load (log10) was 1.9 copies/ml (IQR = 1.6–3.9). According to WHO guidelines [26], 36% of patients were in AIDS stage and 89.8% had received ART treatment. A bivariate analysis revealed statistically significant differences between HIV mono-infection and HIV-HBV coinfection groups in parameters such as sexual orientation, ART treatment and AIDS stage (*P*≤0.05) (Table 1).

**Table 1:** Baseline characteristics of the study population.

Variable	Overall Population (n = 275) n (%)	HIV Mono-infection (n = 242) n (%)	HIV-HBV Coinfection (n = 33) n (%)	P Value
				
Sex				
Male	179 (65.1)	153 (63.2)	26 (78.8)	
Female	96 (34.9)	89 (36.8)	7 (21.2)	0.078^a^
Mean age (SD)	37.4 (11.4)	37.1 (11.7)	39.5 (8.7)	0.258^b^
Sexual orientation				
Heterosexual	181 (65.8)	166 (68.6)	15 (45.5)	
Homosexual	41 (14.9)	33 (13.6)	8 (24.2)	0.050^a^
Bisexual	16 (5.8)	14 (5.8)	2 (6.1)	
Not registered	37 (13.5)	29 (12.0)	8 (24.2)	
Total CD4 cells/mm^3^				
<200	68 (24.7)	57 (23.6)	11 (33.3)	0.222^a^
≥200	207 (75.3)	185 (76.4)	22 (66.7)	
Median (IQR) HIV viral load (log10 copies/ml)	1.9 (1.6–3.9)	1.89 (1.6–3.9)	2.5 (1.6–4.7)	0.070^c^
AIDS stage	99 (36.0)	80 (33.1)	19 (57.6)	0.006^a^
ART treatment	247 (89.8)	220 (90.9)	27 (81.8)	0.053^a^

SD = Standard deviation; IQR = Interquartile range; ART = Antiretroviral therapy.

a Chi square test; ^b^ t-student test; ^c^ U-Mann Whitney test.

The prevalence of HIV-HBV coinfection was 12% (95%CI 8.4–16.4). Among all patients, 11.6% had resolved infection, 3.3% active HBV infection, 5.1% isolated anti-HBc and 8.7% OBI. The immune response to HBV vaccine (titers≥10 U/l) was present in 21.8% of HIV patients (Table 2).

**Table 2:** Prevalence of different categories of HBV status among patients diagnosed with HIV in Bucaramanga, Colombia.

HBV status	n	%	95% CI
			
**Susceptible**	136	49.5	43.5–55.4
**Vaccinated**	60	21.8	16.9–26.7
**Resolved infection**	32	11.6	7.8–15.4
**Active HBV infection**	9	3.3	1.1–5.3
**Isolated Anti-HBc**	14	5.1	2.4–7.7
**OBI**	24	8.7	5.3–12.1
**Total**	275	100	

Multivariate analyses showed that the adjusted prevalence of HIV-HBV coinfection was associated with having AIDS whereas ART treatment had a protective effect against co-infection (Table 3). No differences in clinical parameters were found among participants with HIV-HBV coinfection by OBI status.

**Table 3:** Binomial multivariate model for HIV-HBV coinfection.

Variable	PR*	95% CI
ART treatment	0.4	0.2–1.0
Homosexual or bisexual orientation	1.5	0.7–2.9
AIDS stage	2.7	1.4–5.1

*PR = Prevalence ratio.

**Figure 1:**
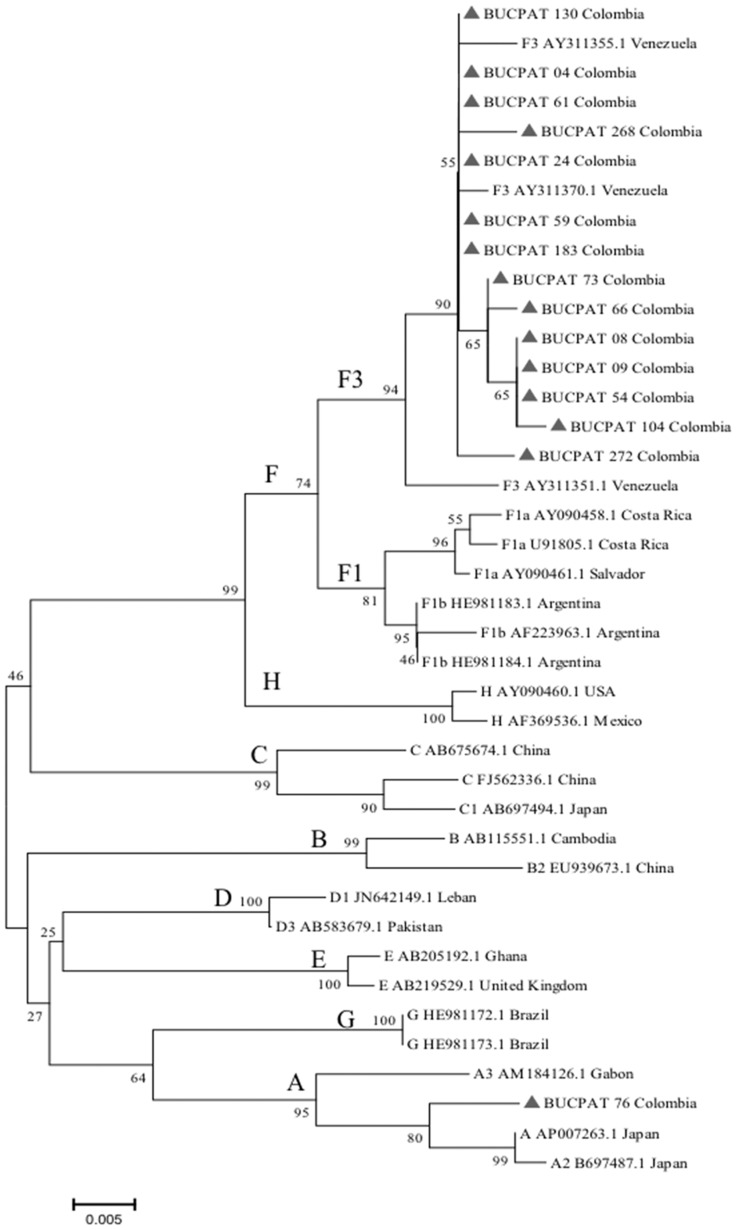
Phylogenetic relationship based on a 425 bp fragment of the small HBs gene (nucleotides 234–659 from EcoRI site) of 15 isolates from HIV patients with OBI (GenBank accession numbers KM583833- KM583847). The phylogenetic tree was constructed using the neighbor-joining method with a 1000-bootstrap replicate analysis indicating the percentage of occurrences (numbers on the nodes). Isolates were portrayed with ▴followed by BUCPAT, the patient's code and Colombia. Each sequence retrieved from the GenBank is designated by the corresponding genotype, accession number and country of origin.

Among 275 HIV-infected patients, HBV-DNA was detected in 30 participants (n = 6 active HBV infection and n = 24 OBI) from which 15 high-quality sequence samples with OBI were obtained for phylogenetic and HBs mutation analysis. A phylogenetic tree based on a 425-nucleotide fragment of the HBV S gene revealed that 14 strains belonged to genotype F subtype F3 (93.3%) and one to genotype A (6.7%) (Figure 1). All HIV patients coinfected with OBI were analysed individually (n = 24) (Table 4). The majority of these patients (75%) were male, average age 39.8 ±9.6 years, and mean CD4 count 313.5 cells/mm^3^, (IQR =  146–458.5). The median HIV viral load (log10) was 2.49 copies/ml (IQR =  1.60–4.78) and 50% were in AIDS stage. Surprisingly, the distribution of HBV serological markers in OBI patients indicated that 12.5% were positive for anti-HBc, 4.2% for anti-HBs, 58.3% were double positive (anti-HBs/anti-HBc) and 25% were negative for all markers. There were three patients with four amino acid substitutions in the MHR (Q101H; C149R; L158G; G159R). We did not detect any mutations in the antigenic “a” determinant of HBsAg (amino acids 124–147).

**Table 4:** HIV and HBV biomarkers in patients coinfected with OBI.

Patient Code	Age	Sex	CD4 counts (cells/ml)	HIV viral load (log10 copies/ml)	AIDS Stage	Positive serological marker for HBV	HBV Genotype	HBsAg changes in the amino acid sequence
4	40	M	428	4,91	No	Anti-HBs/Anti-HBc	F3	wt
5	39	M	339	1,60	No	Anti-HBs/Anti-HBc		
8	47	M	678	1,70	Yes	Anti-HBs/Anti-HBc	F3	wt
9	25	M	210	1,92	Yes	Anti-HBs/Anti-HBc	F3	wt
22	38	M	168	1,60	Yes	None		
24	37	M	144	5,06	No	None	F3	wt
34	21	M	45	4,81	Yes	Anti-HBc		
49	42	M	416	3,51	No	Anti-HBs/Anti-HBc		
53	49	M	219	2,48	Yes	Anti-HBs/Anti-HBc		
54	55	M	600	1,60	No	Anti-HBs/Anti-HBc	F3	G50A
59	54	F	392	3,94	No	Anti-HBs/Anti-HBc	F3	wt
61	47	M	448	1,87	No	Anti-HBs/Anti-HBc	F3	wt
66	45	M	117	2,22	No	Anti-HBs/Anti-HBc	F3	S31T
71	35	F	201	1,60	Yes	Anti-HBs/Anti-HBc		
72	35	M	471	3,87	No	Anti-HBs/Anti-HBc		
73	56	F	576	1,60	Yes	Anti-HBs/Anti-HBc	F3	wt
76	44	M	129	5,65	Yes	Anti-HBs/Anti-HBc	A	S31N/**Q101H**
90	25	F	77	4,74	Yes	None		
104	29	M	148	4,81	No	None	F3	G50A
130	41	F	123	5,23	No	None	F3	**C149R**
183	51	M	668	2,50	No	None	F3	wt
207	33	F	306	1,60	Yes	Anti-HBs		
268	33	M	469	1,60	Yes	Anti-HBc	F3	**L158G/G159R**
272	35	M	153	4,63	Yes	Anti-HBc	F3	wt

M: male; F: female; wt: wild type. Bold case letters correspond to amino acid changes in the MHR of the HBsAg.

## Discussion

The present study is a comprehensive cross-sectional analysis of HBV prevalence in a large group of people living with HIV in northeast Colombia. To the best of our knowledge, this is the first report describing high HIV-OBI coinfection and viral genotypes in the country, regardless of serological HBV markers. Our results confirm the high prevalence of HBV, F3 subgenotype dominance, absence of mutations in the “a” antigen determinant of the HBsAg in OBI patients, and low hepatitis B vaccine coverage, with most HIV-mono-infected individuals susceptible to HBV infection.

The prevalence of HIV-HBV coinfection in HIV patients, defined as HBV exposure and positive results in serological markers of HBV infection, varies worldwide. Mexico and France have reported up to 70% coinfection rates [27], [28]. Coinfection rates in South Africa are around 30%, [29] and 34.7% the United States [30]. Our data showed an overall prevalence (past and current HBV infection) of 28.7%; an estimate that is lower than those of other Latin American countries such as Chile (46.3%), Cuba (45.5%) and Brazil (30–55.1%) [31]–[35]. A previous population-based study conducted in Medellin (Colombia) yielded an HIV-HBV coinfection rate of 38.6% [36] and an active HBV infection rate of 2.1%, which is similar to the 3% prevalence identified by our study in Bucaramanga. Remarkably, the endemicity of HBV mono-infection in the two Colombian cities has been classified as low [19].

Our findings reveal a high (8.7%) prevalence of HIV-OBI coinfection with large heterogeneity in the HBV serological profile. Most previous studies examining OBI in HIV patients have included participants with isolated anti-HBc and other serological markers, with prevalence estimates ranging from 5 to 45% depending on the geographic location, the pattern of HBV endemicity, and the relative sensitivity of viral DNA assays [37], [38]. In this study, we found OBI in 12.5% of patients with isolated anti-HBc; a lower proportion than those reported in Brazil and Mexico (20%) but higher than the 5.6% reported in Argentina [37 39,40]. A number of molecular mechanisms leading to OBI have been proposed including mutations in the “a” determinant, located among amino acids 124–147 of HBsAg, and other viral strategies that impair or reduce HBs gene expression [13]–[15]. We did not find amino acid substitutions in the “a” antigenic loop in patients co-infected with HIV-OBI. Mutations in the MHR (Q101H, C149R, L158G and G159R) were identified in three patients (one of which did not have any hepatitis B serological markers). In a biological model, transfection of HBV genotype A clones in HepG2 cells revealed that the C149R mutation severely impaired HBV secretion in vitro [41]. The mutation G159R has been observed in Iran among HBsAg-positive individuals with genotype D whereas mutation Q101H was found in an OBI patient in India [42], [43]. Taken together, these data confirm that mutations in the “a” antigenic loop or MHR in the HBs gene are not the sole factors associated with OBI.

Another potential mechanism of OBI is suggested by the simultaneous presence of HBV DNA and anti-HBs, implying viral persistence after apparent infection resolution. In the present study, 58.3% of HIV-OBI coinfections were identified as anti-HBs/anti-HBc carriers. Previous findings in Africa have shown incidence rates of an anti-HBs/anti-HBc serological profile of 25% and 33.4% among Nigerian and Ghanaian patients with OBI, respectively. [44], [45]. Our sequence analysis indicated that five anti-HBs/anti-HBc OBI samples were HBV wild-type strains, two of which had amino acid substitutions outside the MHR in the HBs gene. OBI reactivation in the presence of anti-HBs-positive status and past exposure to HBV may cause chronic liver disease among HIV patients [46].

Bivariate analysis revealed differences in sexual orientation, ART treatment and AIDS stage between the HIV mono-infection and HIV-HBV coinfection groups. After adjusting for confounding factors in a multivariate analysis, the prevalence of HIV-HBV coinfection was positively associated with AIDS, whereas ART treatment was found to have a protective effect. The association with AIDS likely represents a higher immunosuppression stage in patients with HIV-HBV co-infection as they had lower CD4 count means, even in the presence of a similar proportion of patients with less than 200 cells/mm^3^. The role of the immune system in the genesis of OBI remains controversial. Some authors have not observed high CD4 counts among HIV-OBI co-infected patients while others have not encountered any association with lower CD4 counts and OBI in HIV settings [47], [49]. The apparent protective effect of ART treatment may be related to a relative immune reconstitution in patients under regular treatment. These findings are in line with a previous report suggesting that OBI should be seen as an opportunistic reactivation of HBV whose replication is likely suppressed by initiation and maintenance of ART treatment [47]

A recent study reported the predominance of male sex and homosexual orientation, possibly due the high risk of exposure to HBV from unprotected sexual intercourse [30]. Likewise, increased HIV-HBV coinfection among older adults has been previously described [7], [48]. On the other hand, low CD4 has been associated with OBI compared to isolated anti-HBc in HIV-coinfected patients (*P* = 0.019) [48]. In contrast to previous studies, our results showed no differences between the HIV mono-infection and HIV-HBV coinfection groups by sex, age or CD4 count.

The phylogenetic tree analysis identified genotype F, subgenotype F3 in most patients (93.8%) and genotype A in 6.2% of patients, as expected for the Colombian population. Previously, in a multi-center blood donors study, Alvarado-Mora et al. characterized F3 strains as the most prevalent subtype in the country (75%) followed by the A2 subgenotype (15.3%) [50].To our knowledge, this is the first report to describe HBV strains among HIV patients in Colombia.

Our study has some limitations. Firstly, even though we enrolled a large HIV-infected population, the small number of OBI and active HBV infection cases precluded the estimation of differences in clinical and molecular parameters between HIV-HBV co-infected subsets. Secondly, we did not sequence viral DNA from HBsAg-positive patients to compare S gene between active HBV and OBI cases. Third, the limited detection of mutations within the “a” determinant likely reflects the use of direct sequence data rather than cloning or no comparison to chronic HBV sequences from the same population. Fourthly, we did not measure alanine aminotransferase (ALT) and aspartate aminotransferase (AST) to evaluate liver injury and its association with HBV infection. Future studies are warranted to analyze clinical, virological and epidemiological variables in a large group of HIV-HBV coinfected patients in Colombia.

In summary, OBI is a public health problem among HIV-infected patients in northeast Colombia that increases the risk of potential complications such as acute liver failure, cirrhosis and CHC. Our results suggest that earlier diagnosis of OBI should include molecular screening of all HIV-positive patients, regardless of their serological HBV pattern. Furthermore, vaccination and anti-HBs antibody screening is recommended to diminish HBV exposure in immune-naïve HIV patients.

## Supporting Information

Text S1 **Supplementary spreadsheet.**
(PDF)
