# Supplementary material for: Epidemiology, Risk Factors and Genotypes of HBV in HIV-Infected Patients in the Northeast Region of Colombia: High Prevalence of Occult Hepatitis B and F3 Subgenotype Dominance
Source: PLoS One. 2014 Dec 2;9(12):e114272. doi: 10.1371/journal.pone.0114272 (PMC4252145; doi:10.1371/journal.pone.0114272)
Supplement: Text S1 — Supplementary spreadsheet. (PDF) [file pone.0114272.s001.pdf]

Text S1: Supplementary Spreadsheet

Appendix Table 1: Demographic, epidemiological, immunological and virologial characteristics of the study population

Patient Code	Age (years)	Sex. 1:Male; 0:Female	Sexual orientation. 0: heterosexual; 1: homosexual; 2:bisexual; 3:No data	CD4 count (cells/mm^3)	HIV viral load (copies/ml)	HAART treatment. No; 1: Yes	AIDS stage 0:No; 1:Yes	HIV treatment (HAART)	HBsAg 0: Negative 1:Positive	Anti-HBc total 0:Negative 1:Positive	Anti-HBs 0:Negative 1:Positive	HBV nested PCR 0: Negatvive; 1:Positive
1	41	1	3	358	40	1	1	Azidotimidina/Lamivudina/Efavirenz	1	1	0	1
2	25	1	3	252	1932741	1	1	Zidovudine/Lopinavir	0	1	1	0
3	60	1	3	511	40	1	0	Lamivudina/Abacavir/Efavirenz	0	1	1	0
4	40	1	3	428	81739	0	0		0	1	1	1
5	39	1	3	339	40	1	0	Lamivudina/Zidovudina/Efavirenz	0	1	1	1
6	46	1	3	235	40	1	0	lamivudina/efavirenz/abacavir	0	1	1	0
7	42	1	3	633	40	1	0	Lamivudina/Zidovudina/Efavirenz	0	1	1	0
8	47	1	3	678	50	1	1	lamivudine/zidovudine/efavirenz	0	1	1	1
9	25	1	3	210	84	1	1	lamivudine /efavirenz/abacavir	0	1	1	1
10	27	1	3	209	69	1	0	lamivudine/zidovudina	0	0	0	0
11	52	1	3	332	40	1	0	Azidotimidina/Lamivudine/Efavirenz	0	0	0	0
12	24	1	3	305	12921	1	0	lamivudine/zidovudina/Efavirenz	0	0	0	0
13	41	1	3	800	40	1	1	Azidotimidina/Lamivudine/Efavirenz	0	0	0	0
14	36	0	3	448	1419	0	0		0	0	0	0
15	18	1	3	776	48	1	1	Lamivudina/Zidovudina/Efavirenz	0	0	0	0
16	49	1	3	368	2749	1	1	Lamivudina/Zidobunina/Efavirenz	0	0	0	0
17	24	1	3	210	84	1	0	Lamivudina/Abacavir/Efavirenz	0	0	0	0
18	35	1	3	664	60	1	0	Lamivudina/Abacavir/ Efavirenz	0	0	0	0
19	28	0	3	734	40	1	0	Lamivudina/Zidovudina/Ritonavir/Lopinavir	0	0	0	0
20	34	1	3	495	1822	0	0		0	0	0	0
21	45	1	3	21	162741	1	0	lamivudina/ritonavir/abacavir	0	0	0	0
22	38	1	3	168	40	1	1	azidovudine/lamivudine	0	0	0	1
23	29	1	3	410	9025	1	0	lamivudina/efavirenz/abacavir	0	0	0	0
24	37	1	3	144	114733	1	0	Lamivudina/Zidovudina/Efavirenz	0	0	0	1
25	24	1	3	478	208	1	0	Lamivudina/Zidovudina/Efavirenz	0	0	0	0
26	15	1	3	250	74	1	0	Lamivudina/Zidovudina/Efavirenz	0	0	0	0

27	44	1	3	77	30539	1	0	lamivudina/zidovudina/atazanavir	0	0	0	0
28	39	1	3	174	37243	1	0	Didanosina, stavudina, ritonavir/lopinavir	0	0	0	0
29	34	1	3	174	5916	1	0	lamivudiva,zidovudina,nelfinavir	0	0	0	0
30	39	0	3	259	40	1	1	Lamivudina/Abacavir/Efavirenz	0	0	1	0
31	9	1	3	422	40	1	0	Lamivudina/Zidovudina	0	0	1	0
32	54	1	3	325	40	1	1	Lamivudina-Abacavir/Ritonavir/Lopinavir	0	0	1	0
33	44	1	3	506	40	1	0	azidotimidina/lamivudina/ritonavir/lopinavir	0	0	1	0
34	21	1	3	45	64410	1	1	Azidptimidina/lamivudine/fluconazol	0	1	0	1
35	28	1	3	77	40	1	0	Lamivudin/Efavirenz	0	1	0	0
36	40	1	3	106	27489	1	0	Lamivudina/Efavirenz	0	1	0	0
37	36	1	3	636	8307	1	0	zidovudina,lamivudina,nevirapina	0	1	0	0
38	43	1	0	250	1086	1	1	Comvivir, Caletra	1	1	0	0
39	37	1	0	437	40	1	1	Lamivudina, Abacavir, Caletra	1	1	0	1
40	41	1	0	185	211067	0	0		1	1	0	1
41	32	1	1	241	4333	1	1	Lamivudina,Zidovudina,Efavirenz	1	1	0	1
42	41	1	1	742	5025	0	0		1	1	0	1
43	43	1	0	178	40	1	1	Lamivudina/Zidovudina, Caletra	0	1	1	0
44	39	0	0	234	40	1	0	Abacavir, Didanosina, Caletra	0	1	1	0
45	41	1	0	237	597	1	1	Lamivudina/Zidovudina, Efavirenz	0	1	1	0
46	31	1	0	659	40	1	1	Lamivudina, abacavir, efavirenz	0	1	1	0
47	45	1	0	265	42	1	1	Lamivudina/Zidovudina, Efavirenz	0	1	1	0
48	47	1	0	185	40	1	1	Lamivudina/Zidovudina, Efavirenz	0	1	1	0
49	42	1	1	416	3217	1	0	Lamivudine/zidovidina/Efavirenz	0	1	1	1
50	44	0	0	26	179392	1	1	Nevirapina, Triavir	0	1	1	0
51	46	0	0	65	40	1	0	Zidobudina, lamivudina, efavirenz	0	1	1	0
52	49	1	2	370	259	1	0	Zidovudina, Lamivudina, efavirenz	0	1	1	0
53	49	1	0	219	305	1	1	lamivudine/Zidovidina/Ritonavir/Lopinavir	0	1	1	1
54	55	1	1	600	40	1	0	Lamivudine/zidovudina/Atazanavir	0	1	1	1
55	45	1	1	26	829007	1	0	Azidotimidina/lamivudine/Efavirenz	0	1	1	0
56	23	1	1	965	40	1	0	Lamivudina/Efavirenz	0	1	1	0
57	47	1	0	489	40	1	0	Zidovudina/Abacavir/Efavirenz	0	1	1	0
58	44	1	1	476	1194	1	0	Azidotimidina/lamivudina/efavirenz	0	1	1	0
59	54	0	0	392	8694	1	0	lamivudine/nelfinavir	0	1	1	1
60	53	0	0	669	241	1	1	lopinavir/ritonavir, abacavir	0	1	1	0

61	47	1	2	448	74	1	0	lamivudina,abacavir,efavirenz	0	1	1	1
62	39	0	0	309	826	1	0	Lamivudina,Zidovudina,nevirapina	0	1	1	0
63	46	1	0	530	274	1	1	Lamivudina, Efavirenz	0	1	1	0
64	54	1	0	359	40	1	0	Lamivudina,Zidovudina,Efavirenz	0	1	1	0
65	32	1	1	783	40	1	0	Lamivudina,zidovudina,lopinavir/ritonavir	0	1	1	0
66	45	1	1	117	165	1	0	lamivudina/stavudina/nevirapina	0	1	1	1
67	55	1	1	286	164	1	0	zidovudina,lamivudina,efavirenz	0	1	1	0
68	43	1	2	212	278	1	0	lamivudine/zidovudine/nevirapine	0	1	1	0
69	35	1	1	110	11290	1	0	Lamivudina/Zidovudina/Efavirenz	0	1	1	0
70	42	1	0	612	40	1	1	zidovudina,lamivudina,lopinavir/ritonavir	0	1	1	0
71	35	0	0	201	40	1	1	lamivudine/efavirenz/azidovudine	0	1	1	1
72	35	1	1	471	7491	0	0		0	1	1	1
73	56	0	0	576	40	1	1	lamivudine/efavirenz/azidovudine	0	1	1	1
74	53	0	0	634	289	1	1	zidovudina,lamivudina,nevirapina	0	1	1	0
75	38	1	0	113	40	1	1	zidovudina,lamivudina,efavirenz	0	1	1	0
76	44	1	0	129	449584	0	1		0	1	1	1
77	58	0	0	41	1220975	1	0	lamivudina/efavirenz/abacavir	0	1	1	0
78	43	1	1	659	40	1	0	azidovudine/lamivudine/efavirenz	0	1	1	0
79	63	1	2	563	67	1	1	azidovidine/lamvudine/ritonavir/lopinavir	0	1	1	0
80	62	1	0	69	54262	1	0	Lamivudina,Zidovudina,Efavirenz	0	1	1	0
81	34	1	0	740	74	1	0	Lamivudina/Zidovudina, Efavirenz	0	0	0	0
82	56	1	0	211	40	1	1	Lamivudina/Zidovudina, Efavirenz	0	0	0	0
83	64	1	0	426	40	1	0	Lamivudina/Zidovudina, Efavirez	0	0	0	0
84	19	0	0	20	9653	1	1	Lamivudina/Zidovudina, Efavirenz	0	0	0	0
85	41	1	0	89	49	1	1	Lamivudina, Abacavir, Caletra	0	0	0	0
86	66	1	2	104	65	1	0	Lamivudina/Zidovudina, Efavirez	0	0	0	0
87	25	1	0	71	10698	1	1	Lamivudina/Zidovudina, Caletra	0	0	0	0
88	34	0	0	243	2581	1	0	Abacavir, Didanosina, Caletra	0	0	0	0
89	39	1	0	496	690	1	1	Lamivudina/Zidovudina, Efavirenz	0	0	0	0
90	25	0	0	77	54658	1	1	Fosamprenavir, Ritonavir, Didanosina, Abacavir	0	0	0	1
91	37	1	0	185	40	1	1		0	0	0	0
92	26	1	0	198	867157	1	1	Azidotimidina/Lamivudine/Efavirenz	0	0	0	0
93	8	1	0	540	40	1	0	Zidovudina, Didanosina, Ritonavir/Lopinavir	0	0	0	0
94	30	1	0	257	18000	1	1	Lamivudina, Stavudina, Caletra	0	0	0	0

95	26	0	0	212	36762	1	0	Abacavir, Didanosina, Caletra	0	0	0	0
96	5	0	0	281	59	1	0	Lamivudina/Zidovudina, Caletra	0	0	0	0
97	23	0	0	353	40	1	1	Lamivudina/Zidovudina, Efavirenz	0	0	0	0
98	34	0	0	270	40	1	1	Azidotimidina/Lamivudine/Ritonavir/Lopanavir	0	0	0	0
99	26	0	0	191	40	1	1	Lamivudina/Zidovudina/Ritonavir/Lopinavir	0	0	0	0
100	43	1	0	273	5500	1	1	Caletra, Stavudina, Abacavir	0	0	0	0
101	38	1	1	87	2426	1	0	Lamivudina/Zidovudina/Efavirenz	0	0	0	0
102	34	0	0	179	40	1	1	Lamivudina/Zidovudina	0	0	0	0
103	43	0	0	263	217	1	1	Lamivudina/Zidovudina, Efavirenz	0	0	0	0
104	29	1	0	148	64678	1	0	Zidobudina, lamivudina, efavirenz	0	0	0	1
105	44	1	2	109	120	1	0	Zidobudina, lamivudina, efavirenz	0	0	0	0
106	35	1	0	152	1131	1	1	Lamivudina, abacavir, efavirenz	0	0	0	0
107	32	0	0	280	40	1	0	Lamivudina, Zidobudina,efavirenz	0	0	0	0
108	65	1	0	637	7691	0	0		0	0	0	0
109	42	1	0	111	40	1	1	Zidobudina, lamivudina, efavirenz	0	0	0	0
110	38	1	0	341	494	1	1	Stavudina, Lamivudina, nelfinavir	0	0	0	0
111	39	0	0	204	1776	1	1	Zidobudina, Lamibudina, Ritonvir/Lopinavir	0	0	0	0
112	43	1	0	554	40	1	0	Azidotimidina/Efavirenz/Lamivudine	0	0	0	0
113	55	1	0	15	1476972	1	0	Lamivudine/Efavirenz/Azidotimidina	0	0	0	0
114	7	1	0	1268	40	1	0	zidovudina,lamivudina,lopinavir/ritonavir	0	0	0	0
115	43	0	0	100	123	1	0	Lamivudine/Efavirenz/Abacavir	0	0	0	0
116	46	0	0	122	4963	1	0	Azidotimidina/Lamivudine/Efavirenz/Lovastatina	0	0	0	0
117	41	0	0	238	784	1	0	Lamivudine/Zidovudina/Efavirenz	0	0	0	0
118	58	1	2	563	11221	0	1		0	0	0	0
119	30	0	0	732	40	1	1	Lamivudina/Efavirenz	0	0	0	0
120	30	1	0	297	37762	1	1	Lamivudina/Zidovudina/Efavirenz	0	0	0	0
121	43	1	0	287	40	1	0	Lamivudina/Abacavir/Nevirapina	0	0	0	0
122	33	0	0	385	40	1	0	Lamivudina/Zidobudina/Efavirenz	0	0	0	0
123	35	1	0	33	1389	1	1	Lamivudina/Zidobunina/Efavirenz	0	0	0	0
124	36	1	0	390	40	1	1	Lamivudina/Zidovudina/Efavirenz	0	0	0	0
125	26	0	0	613	40	1	0	Lamivudina/Zidovudina/Efavirenz	0	0	0	0
126	21	0	0	322	40	1	0	Lamivudina/Zidovudina/Ritonavir/Lopinavir	0	0	0	0
127	36	0	0	393	40	1	0	Azidotimidina/lamivudina/nevirapine/nelfinavir	0	0	0	0
128	42	0	0	523	40	1	0	Lamivudina/Zidovudina/Efavirenz	0	0	0	0

129	23	1	0	157	44251	0	0		0	0	0	0
130	41	0	0	123	170936	0	0		0	0	0	1
131	38	1	0	348	214	1	1	azidovudina/lamivudine/nevirapine	0	0	0	0
132	46	1	0	945	40	1	0	lamivudina,lopinavir/ritonavir	0	0	0	0
133	9	0	0	650	40	1	0	azidovudine/lamivudine/lopinavir/ritonavir	0	0	0	0
134	34	1	0	363	56	1	0	Lamivudina,Zidovudina,Efavirenz	0	0	0	0
135	29	0	0	255	40	1	0	Lamivudina,Zidovudina, Efavirenz	0	0	0	0
136	35	0	0	263	40	1	0	azidovudine/lamivudine/efavirenz	0	0	0	0
137	44	1	0	610	40	1	1	Lamivudina,Zidovudina,Efavirenz	0	0	0	0
138	22	1	1	550	21371	0	1		0	0	0	0
139	44	0	0	16	339515	1	1	Lamivudina,Zidovudina, Lopinavir/Ritonavir	0	0	0	0
140	58	0	0	215	40	1	1	azidovudine/lamivudine/efavirenz	0	0	0	0
141	27	0	0	153	91712	1	0	aazidovudine/lamivudine/efavirenz	0	0	0	0
142	31	0	0	91	14940	1	0	Abacavir/lopinavir/ritonavir	0	0	0	0
143	33	0	0	942	374	0	0		0	0	0	0
144	24	0	0	613	2465	1	0	lamivudine/zidovudine/efavirenz/nelfinavir	0	0	0	0
145	46	1	2	529	40	1	0	lamivudina/zidovudina/efavirenz	0	0	0	0
146	22	0	0	315	86752	0	0		0	0	0	0
147	34	1	0	46	461	1	0	lamivudine/zidovudine/efavirenz	0	0	0	0
148	28	0	0	590	16312	0	0		0	0	0	0
149	37	1	0	201	8615	1	0	zidovudina,lamivudina,nevirapina	0	0	0	0
150	9	0	0	803	40	1	0	lamivudine /zidovudine	0	0	0	0
151	51	0	0	220	40	1	1	Lamivudina,Zidovudina,Efavirenz	0	0	0	0
152	29	1	1	505	13891	0	0		0	0	0	0
153	35	0	0	375	173343	1	0	azidovudine/lamivudine/lopinavir/ritonavir	0	0	0	0
154	27	1	1	331	379	1	0	Lamivudina,Zidovudina,nevirapina	0	0	0	0
155	21	0	0	469	8615	0	0		0	0	0	0
156	40	0	0	153	58	1	0	Lamivudina/Zidovudina/Efavirenz	0	0	0	0
157	24	0	0	232	14349	1	0	Lamivudina/Zidovudina/Efavirenz	0	0	0	0
158	27	0	0	669	40	1	0	didanosina/efavirenz/abacavir	0	0	0	0
159	41	0	0	569	78509	1	0	azidovudine/lamivudine/lopinavir/ritonavir	0	0	0	0
160	55	1	0	140	40	1	0	zidovudina,lopinavir/ritonavir	0	0	0	0
161	43	0	0	795	46	1	0	lamivudina,stavudina,efavirenz	0	0	0	0
162	36	0	1	335	40	0	0		0	0	0	0

163	32	1	1	613	53892	0	0		0	0	0	0
164	60	0	0	133	785377	1	0	lamivudina/zidovudina, ritonavir/lopinavir	0	0	0	0
165	20	1	1	450	800	0	0		0	0	0	0
166	31	0	0	919	839	0	0		0	0	0	0
167	28	0	0	294	40	1	0	lamivudine/zidovudine/nevirapine	0	0	0	0
168	31	1	0	318	91	1	0	lamivudine/abacavir/efavirenz	0	0	0	0
169	28	1	0	685	1225	1	0	zidovudina,lamivudina,nevirapina	0	0	0	0
170	30	1	0	442	8906	1	0	lamivudine/efavirenz/azidovudine	0	0	0	0
171	51	1	0	589	40	1	0	zidovudina,lamivudina,nevirapina	0	0	0	0
172	31	1	0	147	873	1	1	zidovudina,lamivudina,efavirenz	0	0	0	0
173	32	1	1	640	40	1	0	zidovudina,didadosina,lopinavir/ritonavir	0	0	0	0
174	28	1	0	416	40	1	0	zidovudina,lamivudina,efavirenz	0	0	0	0
175	32	1	0	290	10874	1	0	zidovudina,lamivudina,efavirenz	0	0	0	0
176	50	1	0	348	40	1	0	zidovudina,lamivudina,nevirapina	0	0	0	0
177	29	0	0	557	52	1	0	zidovudina,lamivudina,nelfinavir	0	0	0	0
178	58	0	0	136	40	1	0	zidovudina,lamivudina,efavirenz	0	0	0	0
179	28	1	0	547	117828	0	0		0	0	0	0
180	34	1	1	10	3150	1	0	lamivudine/efavirenz/azidovudine	0	0	0	0
181	37	0	0	392	245	1	1	lamivudina,lopinavir/ritonavir,abacavir	0	0	0	0
182	31	1	1	545	40	1	1	lamivudine/zidovudina /nevirapine	0	0	0	0
183	51	1	1	668	319	1	0	lamivudine/zidovudine/atazanabir	0	0	0	1
184	25	1	1	198	116226	1	0	lamivudine/ satvudine/nelfinavir	0	0	0	0
185	23	0	0	534	15372	0	0		0	0	0	0
186	59	0	0	296	8716	0	0		0	0	0	0
187	27	1	0	220	37701	1	1	Raltegravir /lopinavir	0	0	0	0
188	40	1	0	72	73327	1	0	azidovudine/lamivudine/efavirenz	0	0	0	0
189	32	1	1	361	40	1	0	lamivudine/efavirenz/abacavir	0	0	0	0
190	28	1	1	306	29446	1	1	lamivudine/zidovudine/efavirenz	0	0	0	0
191	42	0	0	1087	45	1	0	Lamivudina/Zidovudina/Efavirenz	0	0	0	0
192	40	0	0	1482	40	1	0	azidovudine/lamivudine/efavirenz	0	0	0	0
193	28	1	0	100	106328	1	0	lamivudine/zidovudine/efavirenz	0	0	0	0
194	25	1	1	449	88	1	0	lamivudine/zidovudine/nevirapine	0	0	0	0
195	52	1	0	449	353	1	1	zidovudine/lamivudine/nelfinavir	0	0	0	0
196	43	1	0	32	132989	1	0	lamivudina,efavirenz,abacavir	0	0	0	0

197	39	1	0	183	54110	1	0	Lamivudina/Zidovudina/Nevirapina	0	0	0	0
198	58	0	0	344	39881	1	0	Lamivudina,Zidovudina,Efavirenz	0	0	0	0
199	20	1	0	12	6685	1	0	Lamivudina/Zidovudina/Efavirenz	0	0	0	0
200	36	1	1	57	84	1	1	Lamivudina,zidovudina,lopinavir/ritonavir	0	0	0	0
201	39	0	0	1456	40	1	0	stavudina/didanosina/ritonavir/lopinavir	0	0	0	0
202	45	1	2	314	6950	1	0	lamivudina/efavirenz	0	0	0	0
203	38	0	0	238	50	1	1	abacavir, Videz y Caletra	0	0	1	0
204	44	1	2	482	40	1	0	Caletra, Abacavir, Didanosina	0	0	1	0
205	43	0	0	745	40	1	1	Lamivudina/Zidovudina, Caletra	0	0	1	0
206	36	1	0	502	40	1	1	Lamivudina, Stavudina, Caletra	0	0	1	0
207	33	0	0	306	40	1	1	Lamivudina/Zidovudina, Caletra	0	0	1	1
208	42	1	0	243	40	1	0	Lamivudina/Zidovudina, Efavirenz	0	0	1	0
209	33	0	0	272	40	1	1	Lamivudina, Stavudina, efavirenz	0	0	1	0
210	29	1	1	120	40	1	1	Lamivudina/Zidovudina, Efavirenz	0	0	1	0
211	29	1	0	505	40	1	0	Lamivudina/Zidovudina, Caletra	0	0	1	0
212	45	1	0	217	60334	0	0		0	0	1	0
213	37	0	0	342	14309	1	1	Caletra/Stavudine/Ritonavir/ lopinavir	0	0	1	0
214	51	0	0	479	40	1	1	Lamivudina/Zidovudina, Nelfinavir	0	0	1	0
215	48	1	0	364	299	1	1	Fosamprenavir, Ritonavir, Didanosina, Abacavir	0	0	1	0
216	43	1	2	650	71	1	0	Abacavir, Videz, Caletra	0	0	1	0
217	36	1	1	269	40	1	1	Lamivudina, abacavir, efavirenz	0	0	1	0
218	36	0	0	788	40	1	0	Lamivudina/Zidovudina, Efavirenz	0	0	1	0
219	4	1	0	1493	367	1	0	lamivudina/zidovudina/kaletra	0	0	1	0
220	48	1	0	575	390	1	0	Lamivudina/Stavudina/Efavirenz	0	0	1	0
221	28	1	1	311	40	1	1	Lamivudina/Zidovudina/Efavirenz	0	0	1	0
222	43	1	1	347	377	0	0		0	0	1	0
223	39	1	0	522	40	1	0	Azidotimidina/Lamivudina/Efavirenz	0	0	1	0
224	46	0	0	698	40	1	0	Lamivudina/Abacavir/Efavirenz	0	0	1	0
225	27	0	0	660	40	1	0	lamivudine/azidovudine/efavirenz	0	0	1	0
226	42	1	0	674	40	1	1	Lamivudina/Zidovudina/Ritonavir/Lopinavir	0	0	1	0
227	42	1	1	494	76	1	0	lamivudina/efavirenz/abacavir	0	0	1	0
228	22	0	0	432	2945	0	0		0	0	1	0
229	43	1	1	544	21129	1	1	zidovudina,lamivudina,lopinavir/ritonavir	0	0	1	0
230	4	1	0	607	8956	1	0	Lamivudina/Zidovudina/Ritonavir	0	0	1	0

231	33	0	0	242	56	1	0	Lamivudina,Zidovudina,nevirapina	0	0	1	0
232	32	1	1	482	40	1	0	lamivudina/estavudina/efavirenz	0	0	1	0
233	34	0	0	427	40	1	1	zidovudina,lamivudina,nevirapina	0	0	1	0
234	42	0	0	232	40	1	0	Lamivudina,Zidovudina,nevirapina	0	0	1	0
235	35	0	0	348	159	1	1	lamivudina,abacavir,efavirenz	0	0	1	0
236	40	1	0	333	40	1	1	Lamivudina,zidovudina,lopinavir/ritonavir	0	0	1	0
237	26	0	0	527	40	1	0	lamivudina,abacavir,efavirenz	0	0	1	0
238	47	1	0	468	2151	1	1	Lamivudina,zidovudina,lopinavir/ritonavir	0	0	1	0
239	43	0	0	426	40	1	0	lamivudine/zidovudina/ritonavir/lopinavir	0	0	1	0
240	36	0	0	458	40	1	1	azidovudine/lamivudine/lopinavir/ritonavir	0	0	1	0
241	35	0	0	200	40	1	1	azidovidine/lamvudine/efavirenz	0	0	1	0
242	51	1	0	152	40	1	1	lamivudine/atazanair/abicavir	0	0	1	0
243	46	1	0	361	22917	0	0		0	0	1	0
244	33	0	0	416	40	1	0	lamivudine/zidovudine/efavirenz	0	0	1	0
245	41	1	2	835	40	1	1	Lamivudina,Zidovudina,Efavirenz	0	0	1	0
246	38	0	0	759	40	1	0	lamivudine/zidovudine/efavirenz	0	0	1	0
247	36	0	0	216	1650	1	0	zidovudina,lamivudina,lopinavir/ritonavir	0	0	1	0
248	16	1	0	617	40	1	1	zidovudina,lamivudina,efavirenz	0	0	1	0
249	28	0	0	218	25840	1	0	lamivudina/zidovudina, ritonavir/lopinavir	0	0	1	0
250	38	0	0	371	40	1	0	lamivudine/ abacavir /efavirenz	0	0	1	0
251	27	1	0	351	30331	1	0	zidovudina,lamivudina,efavirenz	0	0	1	0
252	61	1	0	660	40	1	0	zidovuvnina,lamivudina,efavirenz	0	0	1	0
253	32	1	1	29	78	1	0	zidovudina,lamivudina,efavirenz	0	0	1	0
254	38	1	2	887	40	1	0	lamivudine/zidovudina /nevirapine	0	0	1	0
255	35	0	0	813	40	1	1	lamivudina/zidovudine/ritonavir/lopinavir	0	0	1	0
256	59	1	0	414	80	1	1	lamivudine/zidovudine/efavirenz	0	0	1	0
257	41	1	0	1055	40	1	0	azidovudime/klamivudine/atazanavir	0	0	1	0
258	29	1	1	178	45108	1	0	lamivudina/satvudine/lopinavir/ritonavir	0	0	1	0
259	44	0	0	687	40	1	0	lamivudina,zidovudina,nelfinavir	0	0	1	0
260	50	1	2	192	97	1	1	Lamivudina, abacavir, efavirenz	0	1	0	0
261	39	1	0	326	40	1	0	Lamivudine/Efavirenz	0	1	0	0
262	53	1	0	381	40	1	0	Lamivudina/Zidobudina/Nevirapine	0	1	0	0
263	27	0	0	446	209	1	0	Lamivudina/Zidovudina/Ritonavir/Lopinavir	0	1	0	0
264	46	1	2	403	40	1	0	Lamivudina/Zidovudina/Efavirenz	0	1	0	0

265	31	0	0	78	262112	1	0	Azidotimidina/lamivudina/nevirapine/ritonavir/fosampronavir	0	1	0	0
266	40	1	0	0	29058	1	0	lamivudina/efavirenz/abacavir/loperamida	0	1	0	0
267	43	1	0	331	2806	1	0	Lamivudina,Zidovudina,Efavirenz	0	1	0	0
268	33	1	1	469	40	1	1	Lamivudina,Zidovudina,Efavirenz	0	1	0	1
269	50	1	0	315	326	1	1	Stavudina,Lamivudina,Nelfinavir	0	1	0	0
270	38	1	1	381	108625	1	1	zidovudina,lamivudina,lopinavir/ritonavir	0	1	0	0
271	32	1	1	112	81	1	0	lamivudina,lopinavir/ritonavir	0	1	0	0
272	35	1	0	153	42414	1	1	azidovudine/lamivudine /abacavir	0	1	0	1
273	38	1	0	40	97645	1	1	Zidobudina, Lamivudina, Ritonavir/Lopinavir	1	0	0	0
274	28	1	2	342	319	1	1	Didadosina, Lamivudina, Efavirenz	1	0	0	1
275	46	0	0	540	40	1	1	Lamivudina,Zidovudina,Efavirenz	1	0	0	0
